# Tactile, thermal and gustatory stimulation therapy in the treatment of post-stroke oropharyngeal dysphagia: a scoping review

**DOI:** 10.1590/2317-1782/e20230319en

**Published:** 2025-01-20

**Authors:** Julia Matos, Rodrigo Alves de Andrade, Diego Fernando Dornelles Bilheri, Maysa Andrade Santos, Diane de Lima Oliveira, Leandro de Araújo Pernambuco, Ana Maria Furkim

**Affiliations:** 1 Programa de Graduação em Fonoaudiologia, Universidade Federal de Santa Catarina – UFSC - Florianópolis (SC), Brasil.; 2 Departamento de Fonoaudiologia, Universidade Federal de Santa Catarina – UFSC - Florianópolis (SC), Brasil.; 3 Programa de Pós-graduação em Fonoaudiologia, Universidade Federal de Santa Catarina – UFSC - Florianópolis (SC), Brasil.; 4 Departamento de Fonoaudiologia, Universidade Federal da Paraíba – UFPB - João Pessoa (PB), Brasil.

**Keywords:** Deglutition Disorders, Stroke, Stroke Rehabilitation, Taste, Temperature, Therapy

## Abstract

**Purpose:**

To map in the literature the effects of tactile, thermal and/or gustatory stimulation on oropharyngeal dysphagia (OD) post-stroke.

**Methods:**

This scoping review was conducted following the recommendations of PRISMA- ScR and the Joanna Briggs Institute (JBI), registered on the Open Science Framework and developed without language or publication period restrictions. Different databases and grey literature were used for article selection, and the PCC mnemonics constructed the research question ad eligibility criteria, thus including clinical studies involving adults (over 18 years old) diagnosed with OD post-stroke, who received tactile-thermal (TTS) and/or taste-gustatory (TGS) and/or tactile-thermal-gustatory stimulation for treatment, and had their effect measured through examinations, scales, or clinical assessment. The review was conducted blindly and independently by two researchers, and a third was consulted in cases of disagreements.

**Results:**

Three studies were included. None of them addressed an individual TGS protocol, and all presented a TTS protocol. The objectives and evaluation tests of each study were distinct, providing different perspectives about TGS in OD; there was uniformity in the presentation of the applied protocols, frequency, and materials used for therapy.

**Conclusion:**

The studies mapped the effect of TTS from different methodological designs and specific measures; no articles were found that evaluated isolated TGS associated with another technique.

## INTRODUCTION

A stroke is a sudden interruption or reduction of blood flow in the cerebral circulatory pathways, compromising the supply of oxygen and nutrients to cells, caused by a rupture or obstruction of the blood pathway^(1)^. Some factors are associated with its onset, including hypertension, diabetes, high LDL cholesterol and triglycerides, pollution, high body fat, alcoholism, low frequency of physical activity, smoking, and use of other drugs^(2)^.

Dysphagia is one of the symptoms associated with post-stroke complications, characterized by a disorganized sequence of events involving the transport of food, liquids, and fluids from the mouth to the stomach. If left untreated, it can impact the patient's quality of life^(3)^. Dysphagia can lead to dehydration, malnutrition, weight loss, aspiration pneumonia, impaired quality of life, and death^(4)^. Dysphagic patients are approximately 32% more likely to be allocated to hospital sectors with more complex care, and 1.7 times more likely to die in a hospital environment than those without dysphagia^(5)^.

Given the complexity related to dysphagia rehabilitation protocols, Terré^(6)^ shows that different strategies, such as postural maneuvers, diet adaptations, thermal tactile (TTS) and gustatory (TGS) stimulation, neurostimulation, and so forth can be used, according to the patient's needs and the gains from the selected strategy.

Among therapeutic strategies, TTS and TGS aim for greater oral cavity sensitivity and a faster swallowing trigger^(7)^. TTS is traditionally applied with cold touches with a laryngeal mirror, spatula, or spatula wrapped in gauze in the lower third of the palatoglossal arch^(8,9)^.

Sensory stimulation is also related to the increase in cortical activation of swallowing since better responses in the oral and pharyngeal phases due to TTS may be related to cortical reorganization^(10)^.

According to Pelletier and Lawless^(11)^, ingesting citrus-flavored foods substantially increased the number of spontaneous dry swallows. Other studies analyzed TGS and indicated an association of the stimuli with the reduction of pharyngeal transit time^(12,13)^. Studies^(14,15)^ describe changes in swallowing after TTS in dysphagic post-stroke patients. As for non-dysphagic patients, some studies^(16,17)^ did not find significant functional changes in swallowing after TTS.

Thus, TTS and TGS are possible rehabilitation strategies for the post-stroke dysphagic population. However, the different methodological designs, evaluation methods, and outcomes make it difficult to compare and analyze the effectiveness of the therapeutic approach. This study conducted a scoping review to map the studies that evaluate the effect of TTS and TGS in post-stroke dysphagic patients. Such a study design seeks to explore the main findings of the topic in question and ascertain the dimension, scope, and nature of the studies, condensing the data, and making it possible to identify, based on their findings, gaps that direct questions to be answered by systematic reviews and help develop more assertive clinical rehabilitation processes.

The research question was based on the mnemonic elements P (Population), C (Concept), and C (Context), suggested by the JBI guide for scoping reviews. The Population was adults (over 18 years old) diagnosed with oropharyngeal dysphagia (OD) after ischemic stroke; the Concept encompassed the OD treatment with tactile, thermal, and/or oral stimulation, associated or not with another rehabilitation strategy; and the Context was rehabilitation in a hospital, outpatient unit, or rehabilitation center. Therefore, this scoping review aimed to answer the following research question: “How does the scientific literature describe the effects of tactile, thermal, and/or gustatory stimulation in post-stroke dysphagic patients treated in rehabilitation centers, hospitals, or outpatient units?”.

## METHOD

### Protocol and registry

The protocol of this scoping review was registered in the Open Science Framework under registry DOI 10.17605/OSF.IO/QNDKC.

### Writing guide

This scoping review followed the recommendations of the Preferred Reporting Items for Systematic Reviews and Meta-Analyses – Extension for Scoping Reviews (PRISMA-ScR)^(18)^ and the JBI Manual for Evidence Synthesis^(19)^.

A priori conceptual definitions were made to prepare this scoping review:

*Ischemic stroke:* A change in blood circulation resulting from an obstructive phenomenon, compromising the passage of blood through the cerebral circulatory pathways^(1)^;
*Swallowing:* The orderly physiological process of transferring saliva or food contents from the mouth to the stomach^(20)^;
*Dysphagia:* A pathological difficulty in swallowing^(21)^;
*Swallowing rehabilitation:* achieving nutritional stability to avoid laryngotracheal aspiration and its consequences^(22)^;
*Effect*: Defined as the combination between efficiency^(23)^, effectiveness^(24)^, and safety^(25)^;
*Thermal tactile stimulation (TTS):* Therapeutic technique that uses touch (through a laryngeal mirror) and temperature (especially cold temperature) to stimulate oral sensitivity^(26)^;
*Thermal gustatory stimulation (TGS):* Therapeutic technique that uses temperature and taste stimuli to reduce pharyngeal transit time^(27)^.

### Inclusion criteria

The inclusion criteria for this review were studies that address the implementation of treatment with tactile, thermal, and/or gustatory stimulation, associated or not with other rehabilitation strategies, in adults (aged over 18 years) diagnosed with OD resulting from ischemic stroke and who underwent clinical and/or instrumental swallowing assessment before and after treatment. Studies of different designs were selected, without restrictions on language and publication time.

### Exclusion criteria

The review excluded articles that did not describe the pre- and post-therapy monitoring criteria to measure the results; that analyzed the effectiveness of TTGS without separating the types of strokes; without abstracts; unavailable for full-text reading after requesting the authors; literature reviews; duplicate publications; letters; books; conferences; conference abstracts; opinion articles; guides; technical articles; editorial letters; and non-peer-reviewed literature.

### Sources of information

Electronic health databases (CINAHL, Cochrane, EMBASE, LILACS, PEDro, PubMed/MEDLINE, PsycINFO, SciELO, Scopus, and Web of Science) and the gray literature (Google Scholar) were searched on September 13, 2023. A new search was carried out on April 15, 2024, to map possible studies for inclusion.

### Search strategy

The search strategy was based on appropriate combinations of keywords selected in a query to the Health Sciences Descriptors (DeCS)^(28)^ and the Medical Subject Headings (MeSH)^(29)^. Free terms were used if they were considered appropriate. The search strategy per database is presented in Chart 1.

**Chart 1:** Description of search strategies and the number of studies identified during the selection phase

**Databases**	**Search Strategy**	**Studies Identified**
PubMed/MEDLINE	((“Stroke”[Mesh] OR “Stroke” OR “Apoplexy” OR “Brain Vascular Accident” OR “Brain Vascular Accidents” OR “CVA” OR “CVAs” OR “Cerebrovascular Accident” OR “Cerebrovascular Accidents” OR “Strokes” OR “Cerebral ictus”) AND (“Deglutition Disorders”[Mesh] OR “Deglutition Disorders” OR “Dysphagia” OR “Swallowing Disorder” OR “Swallowing Disorders” OR “Deglutition”[Mesh] OR “Deglutition”) AND (“Touch”[Mesh] OR “Touch” OR Tacti* OR “Thermosensing”[Mesh] OR “Thermosensing” OR “Thermosensings” OR “Thermic Treatment” OR “Cold” OR “Temperature” OR Thermal* OR Thermic* OR “Taste Perception”[Mesh] OR “Taste Perception” OR Gustator* OR “Taste”) AND (“Therapeutics”[Mesh] OR “Therapeutics” OR Therap* OR Treatment* OR “Rehabilitation”[Mesh] OR “Rehabilitation” OR Rehabilita*))	55
EMBASE	((“Stroke” OR “Apoplexy” OR “Brain Vascular Accident” OR “Brain Vascular Accidents” OR “CVA” OR “CVAs” OR “Cerebrovascular Accident” OR “Cerebrovascular Accidents” OR “Strokes” OR “Cerebral ictus”) AND (“Deglutition Disorders” OR “Dysphagia” OR “Swallowing Disorder” OR “Swallowing Disorders” OR “Deglutition”) AND (“Touch” OR Tacti* OR “Thermosensing” OR “Thermosensings” OR “Thermic Treatment” OR “Temperature” OR Thermal* OR Thermic* OR “Taste Perception” OR “Cold” OR Gustator* OR “Taste”) AND (“Therapeutics” OR Therap* OR Treatment* OR “Rehabilitation” OR Rehabilita*))	304
CINAHL	((“Stroke” OR “Apoplexy” OR “Brain Vascular Accident” OR “Brain Vascular Accidents” OR “CVA” OR “CVAs” OR “Cerebrovascular Accident” OR “Cerebrovascular Accidents” OR “Strokes” OR “Cerebral ictus”) AND (“Deglutition Disorders” OR “Dysphagia” OR “Swallowing Disorder” OR “Swallowing Disorders” OR “Deglutition”) AND (“Touch” OR Tacti* OR “Thermosensing” OR “Thermosensings” OR “Cold” OR “Temperature” OR “Thermic Treatment” OR Thermal* OR Thermic* OR “Taste Perception” OR Gustator* OR “Taste”) AND (“Therapeutics” OR Therap* OR Treatment* OR “Rehabilitation” OR Rehabilita*))	29
Cochrane Library	((“Stroke” OR “Apoplexy” OR “Brain Vascular Accident” OR “Brain Vascular Accidents” OR “CVA” OR “CVAs” OR “Cerebrovascular Accident” OR “Cerebrovascular Accidents” OR “Strokes” OR “Cerebral ictus”) AND (“Deglutition Disorders” OR “Dysphagia” OR “Swallowing Disorder” OR “Swallowing Disorders” OR “Deglutition”) AND (“Touch” OR Tacti* OR “Thermosensing” OR “Thermosensings” OR “Cold” OR “Temperature” OR “Thermic Treatment” OR Thermal* OR Thermic* OR “Taste Perception” OR Gustator* OR “Taste”) AND (“Therapeutics” OR Therap* OR Treatment* OR “Rehabilitation” OR Rehabilita*))	69
Scopus	((“Stroke” OR “Apoplexy” OR “Brain Vascular Accident” OR “Brain Vascular Accidents” OR “CVA” OR “CVAs” OR “Cerebrovascular Accident” OR “Cerebrovascular Accidents” OR “Strokes” OR “Cerebral ictus”) AND (“Deglutition Disorders” OR “Dysphagia” OR “Swallowing Disorder” OR “Swallowing Disorders” OR “Deglutition”) AND (“Touch” OR Tacti* OR “Thermosensing” OR “Thermosensings” OR “Cold” OR “Temperature” OR “Thermic Treatment” OR Thermal* OR Thermic* OR “Taste Perception” OR Gustator* OR “Taste”) AND (“Therapeutics” OR Therap* OR Treatment* OR “Rehabilitation” OR Rehabilita*))	145
Web of Science	((“Stroke” OR “Apoplexy” OR “Brain Vascular Accident” OR “Brain Vascular Accidents” OR “CVA” OR “CVAs” OR “Cerebrovascular Accident” OR “Cerebrovascular Accidents” OR “Strokes” OR “Cerebral ictus”) AND (“Deglutition Disorders” OR “Dysphagia” OR “Swallowing Disorder” OR “Swallowing Disorders” OR “Deglutition”) AND (“Touch” OR Tacti* OR “Thermosensing” OR “Thermosensings” OR “Cold” OR “Temperature” OR “Thermic Treatment” OR Thermal* OR Thermic* OR “Taste Perception” OR Gustator* OR “Taste”) AND (“Therapeutics” OR Therap* OR Treatment* OR “Rehabilitation” OR Rehabilita*))	82
LILACS	((“Acidente Vascular Cerebral” OR “AVC” OR “AVE” OR “Acidente Cerebral Vascular” OR “Acidente Cerebrovascular” OR “Acidente Vascular Encefálico” OR “Acidente Vascular do Cérebro” OR “Acidentes Cerebrais Vasculares” OR “Acidentes Cerebrovasculares” OR “Acidentes Vasculares Cerebrais” OR “Apoplexia” OR “Derrame Cerebral” OR “Icto Cerebral” OR “Ictus Cerebral” OR “Accidente Cerebrovascular” OR “ACV” OR “Accidente Cerebral Vascular” OR “Accidente Cerebrovascular” OR “Accidente Vascular Cerebral” OR “Accidente Vascular Encefálico” OR “Accidente Vascular del Cerebro” OR “Accidentes Cerebrovasculares” OR “Apoplejía” OR “Ataque Cerebral” OR “Ataque Cerebrovascular” OR “Stroke” OR “Apoplexy” OR “Brain Vascular Accident” OR “Brain Vascular Accidents” OR “CVA” OR “CVAs” OR “Cerebrovascular Accident” OR “Cerebrovascular Accidents” OR “Strokes” OR “Cerebral ictus”) AND (“Transtornos de Deglutição” OR Disfagia* OR “Deglutição” OR “Trastornos de Deglución” OR “Deglución” OR “Deglutition Disorders” OR “Dysphagia” OR “Swallowing Disorder” OR “Swallowing Disorders” OR “Deglutition”) AND (“Tato” OR “Tátil” OR “Táteis” OR “Sensação Térmica” OR “Tratamento Térmico” OR “Estimulação gustativa” OR “Estimulação tátil-térmica” OR Térmic* OR “Percepção Gustatória” OR Gustativ* OR “Tacto” OR “Sensación Térmica” OR “Tratamiento Térmico” OR “Percepción del Gusto” OR “Gusto” OR “Temperatura” OR “Touch” OR Tacti* OR “Thermosensing” OR “Thermosensings” OR “Cold” OR “Temperature” OR “Thermic Treatment” OR Thermal* OR Thermic* OR “Taste Perception” OR Gustator* OR “Taste”) AND (“Terapêutica” OR Terapêutica* OR Terapia* OR Tratamento* OR “Reabilitação” OR Reabilita* OR Tratamiento* OR “Rehabilitación” OR “Therapeutics” OR Therap* OR Treatment* OR “Rehabilitation” OR Rehabilita*))	6
PEDro	Stroke* Dysphag*	128
SciELO	((“Acidente Vascular Cerebral” OR “AVC” OR “AVE” OR “Acidente Cerebral Vascular” OR “Acidente Cerebrovascular” OR “Acidente Vascular Encefálico” OR “Acidente Vascular do Cérebro” OR “Acidentes Cerebrais Vasculares” OR “Acidentes Cerebrovasculares” OR “Acidentes Vasculares Cerebrais” OR “Apoplexia” OR “Derrame Cerebral” OR “Icto Cerebral” OR “Ictus Cerebral” OR “Accidente Cerebrovascular” OR “ACV” OR “Accidente Cerebral Vascular” OR “Accidente Cerebrovascular” OR “Accidente Vascular Cerebral” OR “Accidente Vascular Encefálico” OR “Accidente Vascular del Cerebro” OR “Accidentes Cerebrovasculares” OR “Apoplejía” OR “Ataque Cerebral” OR “Ataque Cerebrovascular” OR “Stroke” OR “Apoplexy” OR “Brain Vascular Accident” OR “Brain Vascular Accidents” OR “CVA” OR “CVAs” OR “Cerebrovascular Accident” OR “Cerebrovascular Accidents” OR “Strokes” OR “Cerebral ictus”) AND (“Transtornos de Deglutição” OR Disfagia* OR “Deglutição” OR “Trastornos de Deglución” OR “Deglución” OR “Deglutition Disorders” OR “Dysphagia” OR “Swallowing Disorder” OR “Swallowing Disorders” OR “Deglutition”) AND (“Tato” OR “Tátil” OR “Táteis” OR “Sensação Térmica” OR “Tratamento Térmico” OR “Estimulação gustativa” OR “Estimulação tátil-térmica” OR Térmic* OR “Percepção Gustatória” OR Gustativ* OR “Tacto” OR “Sensación Térmica” OR “Tratamiento Térmico” OR “Percepción del Gusto” OR “Gusto” OR “Temperatura” OR “Touch” OR Tacti* OR “Thermosensing” OR “Thermosensings” OR “Cold” OR “Temperature” OR “Thermic Treatment” OR Thermal* OR Thermic* OR “Taste Perception” OR Gustator* OR “Taste”) AND (“Terapêutica” OR Terapêutica* OR Terapia* OR Tratamento* OR “Reabilitação” OR Reabilita* OR Tratamiento* OR “Rehabilitación” OR “Therapeutics” OR Therap* OR Treatment* OR “Rehabilitation” OR Rehabilita*))	2
PsycINFO	((“Stroke” OR “Apoplexy” OR “Brain Vascular Accident” OR “Brain Vascular Accidents” OR “CVA” OR “CVAs” OR “Cerebrovascular Accident” OR “Cerebrovascular Accidents” OR “Strokes” OR “Cerebral ictus”) AND (“Deglutition Disorders” OR “Dysphagia” OR “Swallowing Disorder” OR “Swallowing Disorders” OR “Deglutition”) AND (“Touch” OR Tacti* OR “Thermosensing” OR “Thermosensings” OR “Cold” OR “Temperature” OR “Thermic Treatment” OR Thermal* OR Thermic* OR “Taste Perception” OR Gustator* OR “Taste”) AND (“Therapeutics” OR Therap* OR Treatment* OR “Rehabilitation” OR Rehabilita*))	5
Google Scholar	Portuguese:	300
	(“Acidente Vascular Cerebral” OR “AVC” OR “AVE” OR “Acidente Vascular Encefálico”) AND (Disfagia* OR “Deglutição”) AND (“Tato” OR “Tátil” OR Táteis OR Térmic* OR “Gustatória” OR Gustativ*) AND (Terapêutica* OR Terapia* OR Tratamento* OR Reabilita*)	
	Spanish:	
	(“Accidente Cerebrovascular” OR “ACV” OR “Accidente Vascular Encefálico”) AND (Disfagia* OR “Deglución”) AND (“Tacto” OR Térmic* OR “Gusto” OR “Temperatura”) AND (Terapia* OR Tratamiento* OR “Rehabilitación”)	
	English:	
	(“Stroke” OR “Strokes” OR “CVA” OR “CVAs” OR “Cerebrovascular Accident”) AND (“Dysphagia” OR “Swallowing Disorder” OR “Deglutition”) AND (“Touch” OR Tacti* OR “Thermosensing” OR Thermal* OR Thermic* OR Gustator* OR “Taste”) AND (Therap* OR Treatment* OR Rehabilita*)	

### Evidence selection process

The reviewers were calibrated to ensure that text evaluation would follow the pre-established criteria and that analyses would have a standard of agreement. The Kappa coefficient of agreement was applied as a quality-of-fit measure to provide higher-quality data to better assess agreements and divergences during the calibration. Hence, the calibration led to an almost perfect level of coefficient of agreement between the reviewers (Kappa > 90).

A standard examiner (M.A.S), who guided the research and use of the Rayyan Systematic management platform^(30)^, conducted the entire process of training and calibrating the independent reviewers, both speech-language-hearing pathologists (D.F.D.B/J.M). They were trained with 42 articles; afterward, the independent reviewers (D.F.D.B/J.M) performed the steps blindly. Decisions on possible disagreements were resolved by consensus to guarantee the quality of the processes and with the participation of the third reviewer (A.M.F) when necessary.

All identified articles were included in Rayyan Systematic and analyzed after calibration. Two independent, blind reviewers (D.F.D.B/J.M) selected the articles in two stages. In the first one, they analyzed the titles and abstracts of the citations collected in the databases and selected potentially relevant studies. In the second stage, they read the articles in full according to the eligibility criteria and excluded those that did not meet the criteria.

Decisions on potential conflicts were resolved among the reviewers by consensus to ensure the quality of the processes and with the participation of the third reviewer (A.M.F) when necessary.

### Data extraction

The review included studies that performed clinical and/or instrumental evaluation of swallowing before and after treatment to increase the reliability of the extracted data on TTS and TGS. It also collected mean, standard deviation, and p-value data to compare pre- and post-treatment results. Two evaluators extracted all data independently and blindly, and a third evaluator was consulted when necessary.

The following data were extracted: authorship, year, country, study design, study objective, diagnostic data (National Institutes of Health Stroke Scale [NIHSS], time since stroke onset, and location of assessment), sample profile (number of participants, age, sex, and location/side of ischemic stroke), information on professionals responsible for therapy, clinical or instrumental tests for pre and post-assessment, TTS, TGS, or combined treatment protocols, results, and, lastly, possible adverse events.

#### Summary of results

The database search results were organized in a flowchart, and the extracted data were organized in a table, according to classifications established by the judges. When data were quantitatively unavailable in the studies, they were summarized from the description in the body of the text of the articles.

## RESULTS

**Figure 1:**
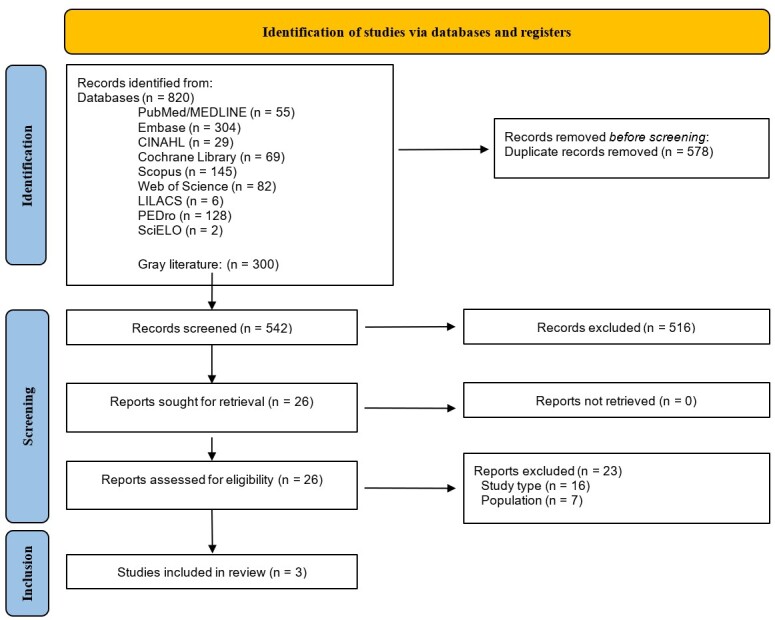
Study selection flowchart

The study selection flowchart is shown in Figure 1. Altogether, 820 studies were identified in the databases and, after removing duplicates, the titles and abstracts of the remaining 542 studies were read. Twenty-six studies were eligible for full reading during phase two, and three of these were included in this scoping review^(31-33)^.

They were published in 1991^(31)^, 1996^(32)^, and 2021^(33)^, the latter in South Korea^(33)^ and the other two in the United States^(31,32)^. Regarding study designs, the first is a single-subject experimental study (ABAB design)^(31)^, replicated with seven other subjects; the second is a crossover study^(32)^, and the third is a randomized controlled experimental study^(33)^.

The studies aimed to verify the thermal influence on swallowing, whether the influence would last, and whether baseline tests could predict the subject's response to thermal stimulation^(31)^; to present data on the variable duration of swallowing and the short-term effects of thermal application^(32)^; and to verify the effect of stimulation with low-temperature capsaicin on dysphagia, feeding level, risk of aspiration or penetration, and nutritional status^(33)^.

Regarding the clinical data in the studies, two of them included patients with a history of multiple strokes^(31,32)^; two presented NIHSS data^(31,33)^; one article did not present the time of stroke onset^(33)^, one presented it in days^(31)^, and another in weeks^(32)^; and only two articles reported where the evaluations took place^(32,33)^.

The studies analyzed predominantly male samples, with n ranging from seven to 43 participants. Two studies used intragroup analysis^(31,32)^, and one used intergroup analysis^(33)^. One article classified the stroke based on the affected side^(33)^ (with a higher percentage on the right side in the intervention group and on the left for the control group), while the others described it based on the affected location^(31,32)^.

Regarding the swallowing assessment, two articles performed an instrumental assessment with a videofluoroscopic swallowing study (VFSS)^(31,32)^, and one article used the Gugging Swallowing Screen (GUSS) for the clinical swallowing assessment and the Measurement System Swallowing Scale (ASHA-NOMS) to assess the dietary level^(33)^.

None of the studies presented specific information about the training and experience of the professionals responsible for the therapy^(31-33)^.

All three articles described the materials used and the frequency of application. They also presented the procedure protocol: two articles applied a size 00 chilled laryngeal mirror to the anterior pillars of the fauces, three times on each side, and then requested the swallowing of a given volume^(31,32)^; the third article applied a solution of capsaicin, water, and thickener to the oropharyngeal mucosa, using a tongue depressor wrapped in gauze, with subsequent application of the capsaicin solution to the oral cavity^(33)^. All of them addressed TTS^(31-33)^, but none approached TGS. One article reported the absence of adverse effects from the intervention^(33)^, and two articles did not report possible events^(31,32)^.

Table 1 presents the sample data, protocol, evaluation data, and results found in the selected studies, all referring to TTS therapy.

**Table 1:** Data extracted from selected studies

Author, year (country)	Study design	Study objective	Diagnosis (NIHSS data, time since stroke onset, location of the assessment)	Sample profile		Tests to assess swallowing and used during Treatment	Professional responsible for the therapy	TTS or TGS protocol	Combined treatment	Results	Adverse events
				Intervention group	Control group						
Rosenbek et al.^(31)^ 1991 (USA)	Single-subject experimental design (ABAB) with replication in 7 other subjects	1: Does daily thermal stimulation influence liquid swallowing? 2: Do the influences persist one month after the end of treatment? 3: Can baseline tests predict the response to thermal stimulation?	Multiple ischemic strokes; NIHSS 9 to 23; Time since stroke: most recent 19 days, oldest reported 5110 days; Location: NR	**7 men; Age** 65 to 77 years; **Location of the stroke** 2 in pons, 1 in cerebellum, 2 in left middle cerebral artery, 2 in right middle cerebral artery		**VFSS**: once pre-therapy, four times during therapy (1x/week), and once one month post-therapy	Clinician	Application of a chilled laryngeal mirror, size 00, to the anterior pillars of the fauces, three times on each side. Afterwards, the participant swallowed 3 cc of water using a spoon or pipette or received a small volume of ice chips.	NR	**Duration measures: OPBM** (s) (mean-SD): 0.15/0.43 - 0.27/0.73 OPBM (s) (mean-SD) **HBRPBMR** (s) (mean-SD): 0.03/0.08 - 0.06/0,10; **BMHE** (s) (mean-SD): 0.03/0.08 - 0.06/0.06; **FMHE** (s) (mean-SD): 0.02/0.11 - 0.02/0.12; **LMMHE** (s) (mean-SD): 0.03/0.22 - 0.05/0.19; **MAHP** (s) (mean-SD): 0.02/0.03 - 0.02/0.04; **LMMAHP** (s) (mean-SD): 0.02/0.08 - 0.02/0.05; **HRR** (s) (mean-SD): 0.10/0.16 - 0.019/0.20; **UESO** (s) (mean-SD): 0.01/0.03 - 0.02/0.02; **UESC** (s) (mean-SD): 0.03/0.06 - 0.03/0.06; **BLUES** (s) (mean-SD): 0.03/0.05 - 0.03/0.04. **DST 1^st^/2^nd^/3^rd^ swallow (s): Subject 1:** 0.72/0.61/1.80; **Subject 2:** 0.53/1.37/0.25; **Subject 3:** 0.51/-0.17/9.64; **Subject 4:** 0.33/0.22/0.56; **Subject 5:** 2.28/0.65/0.20; **Subject 6:** -0.38/0.29/0.41; **Subject 7:** 0.18/-0.13/-; **DST per group, (s): 63-67 years: Mean (SD):** -0.07 (0.27); **70-76 years: Mean (SD):** 0.11 (0.45).	NR
Rosenbek et al.^(32)^ 1996 (USA)	Crossover study	To provide data on variability in objectively measured durational swallowing parameters performed by dysphagic patients secondary to stroke, and to examine the short-term effects of thermal application on these same durational measures.	Multiple ischemic strokes NIHSS NR Time since stroke: < 1 to 172 weeks Location: Hospital	**22 participants;** Age 55 to 81 years (mean of 67.3). **Location of stroke** 2 in the cerebellum, 5 in the posterior limb of the internal capsule, 3 in the pons, 3 in left pontomesencephalic junction, 5 in basal ganglia, 4 in the left parietal lobe.		**VFSS:** during the 10 required swallows	Experienced clinician	Application of a chilled laryngeal mirror, size 00, to the anterior pillars of the fauces, three times on each side. Stimulation on each side was performed in less than 6 seconds after removing the mirror from the ice. After stimulation, patients swallowed a bolus.	NR	**DST: Untreated: Mean (SD):** 1.25 (0.47) **Median:** 0.42 **Treated: Mean (SD):** 0.89 (0.38) **Median:** 0.38 **Difference: Mean (SD):** -0.35 (0.19) **Median:** -0.15 ***p*** 0.046; **TSD: Untreated: Mean (SD):** 2.96 (.50) **Median:** 2.22 **Treated: Mean (SD):** 2.44 (.40) **Median:** 2.00 **Difference: Mean (SD):** -0.52 (.20) **Median:** -0.22 ***p*** 0.005	NR
Bae and Lee^(33)^ 2021 (Korea)	Randomized controlled before-and-after experimental study	To determine the effect of oropharyngeal sensory stimulation with low-temperature capsaicin on swallowing disorder, feeding level, aspiration pneumonia, and nutritional status in patients admitted to the stroke intensive care unit with dysphagia.	Mean NIHSS (SD) - 9.65 (6.52); Time of stroke - NR; 40 Hospital Assessments, 3 Outpatient Assessments	**21 participants; Age** 70.76 (9.19); **Men** 11 (52.4%), **Women** 10 (47.6%); **Side of ischemic stroke: Left** 8 (38.1); **Right** 12 (57.1); **Bilateral** 0; **Multiple** 1 (4.8)	**22 participants; Age** 72.86 (11.83); **Men** 13 (59.1%), **Women** 9 (40.9%); **Side of the ischemic stroke: Left** 12 (54.5), **Right** 6 (27.3), **Bilateral** 4 (18.2), **Multiple** 0	**GUSS:** 1 time before the intervention and on the 3rd and 7th days of intervention. The level of dysphagia was assessed; **ASHA-NOMS:** used to assess dietary level before therapy and on days 3 and 7 of intervention.	NI	2 g of capsaicin powder was diluted in 100 mL of bottled water - a solution with a concentration of 150 uM/L. A thickener (Viscoup) was included, one packet per 100 mL. A tongue depressor wrapped in gauze was soaked in the solution and the researcher gently and quickly rubbed the oropharyngeal mucosa, lightly tapping the area. Afterwards, 1 mL of capsaicin solution was requested in a 2 cc syringe, the needle was removed, and the contents were applied to the patient's oral cavity. The intervention was applied twice a day before meals, for 7 days. The control group received only water, without the addition of capsaicin, at the same time, method, and number of times.	NR	**Dysphagia: Experimental group: Start:** 11.76±4.84; **3^rd^ day:** 14.14±5.23; **7^th^ day:** 16.38±4.84 (χ^2^ = 34.74. p < 0.001)**; Control group: Start:** 10.95±7.20**; 3^rd^ day:** 11.68±6.85; **7^th^ day:** 12.59±7.08 (χ^2^ = 5.41. p = 0.067); **Dietary level: experimental group: Start:** 3.43 ± 1.47; **3^rd^ day:** 4.19±1.81; **7^th^ day:** 4.81±1.81 (χ^2^ = 25.29. p < 0.001). **Control group: Start:** 3.36±2.15; **3^rd^ day:** 3.45±1.81; **7^th^ day:** 3.77±2.22 (χ^2^ = 4.51. p = .105); **Aspiration pneumonia: 3^rd^ day:** no case reported; **7^th^ day: Control group:** 6 (27.3%); **Experimental group:** 2 (9.5%); Incidence rate with no clear difference between groups (x^2^ = 2.23. p = 0.240).	No adverse events

Caption: *NIHSS* = stroke scale; *TTS* = thermal tactile stimulation; *TGS* = thermal gustatory stimulation; *NR* = not reported; *VFSS* = videofluoroscopic swallowing study; *GUSS* = Gugging Swallowing Screen; *ASHA-NOMS* = Measurement System Swallowing Scale; *DST* = duration of stage transition; *TSD* = total swallowing duration; *OPBM* = onset of posterior bolus movement; *HBRPBMR* = The head of the bolus reaches the posterior border of the mandibular ramus; *BMHE* = beginning of maximum hyoid elevation; *FMHE* = first maximum hyoid elevation; *LMMHE* = last moment of maximum hyoid elevation; *MAHP* = most anterior hyoid position; *LMMAHP* = last moment of the most anterior hyoid position; *HRR* = Hyoid returns to rest; *UESO* = upper esophageal sphincter opens; *UESC* = upper esophageal sphincter closes; *BLUES* = the bolus leaves the upper esophageal sphincter; *SD* = standard deviation; *x^2^* = chi-square test; *p* = p-value; *cc* = cubic centimeters; *g* = grams; *s* = seconds; *mL* = milliliters; *µM/L* = micrometer/liter

## DISCUSSION

TTS and TGS approaches are frequently used to treat patients with OD after ischemic stroke. Understanding the effects of such techniques is essential to ensure interventions that actually improve the patient's condition. Thus, this scoping review aimed to verify the effects of TTS and TGS therapies and present an overview of studies that address these techniques in adults with ischemic stroke and dysphagia treated in rehabilitation centers, hospitals, and outpatient clinics.

None of the selected studies addressed TGS therapy alone or in association with TTS. Hence, all three addressed TTS alone^(31-33)^.

Different studies address TTS and TGS among the therapeutic possibilities for OD. However, studies analyzing heterogeneous samples in the same analysis group, with ischemic and hemorrhagic stroke^(34-38)^, predominated throughout the article selection process. This characteristic was found in both the control and intervention groups^(34,36)^.

Silva^(39)^ analyzed the efficiency and efficacy of various therapeutic procedures and criticized the low specificity in samples related to neurogenic dysphagia. Each disease has different involvements – without a homogeneous sample, evidence collection lacks robustness. This criticism is in line with the data obtained in this review since studies were excluded because they evaluated the effect of TTS/TGS considering ischemic and hemorrhagic stroke in the same comparative sample. Therefore, considering the variation in the involvement of ischemic and hemorrhagic stroke related to dysphagia and their frequency^(40,41)^, both populations should be approached independently.

The studies included in the review had different objectives. This influenced several factors in the methodology, with emphasis on the variation in the articles’ days of intervention and therapeutic plan: one of the studies analyzed the influence of daily TTS on the swallowing of liquids and verified the swallowing pattern in the long term (1 month)^(31)^; another article analyzed the effects of TTS in the short term^(32)^; and the third article aimed to determine the effects of applying low-temperature capsaicin in four aspects – dysphagia, dietary level, aspiration pneumonia, and nutritional status^(33)^. TTS applications can verify various perspectives. Hence, despite the few studies in the review, it obtained information and identified gaps for further investigation in future research.

The sample size in these studies ranged from seven to 43 participants, highlighting the need for studies with numerically more representative populations to achieve more robust results. Ischemic stroke affects approximately 7.6 million people per year worldwide^(42)^, and one of the main sequelae is OD, which can affect approximately 50% of individuals^(43)^.

Furthermore, the studies did not standardize, include in their entirety, or report other important data about the sample, such as the time since stroke onset, percentage of males and females in the groups, region affected by the ischemic stroke, NIHSS values, ​​and location of the assessments. In general, they also did not describe information regarding the training and practical experience of the professionals who performed the assessment and therapy. Such data may compromise the comparability between studies, the analysis of the information, its interpretation by other researchers, and the validity of the results.

Another relevant aspect is the choice of different tests to assess and analyze the effects on OD. The different classification methods were based on imaging^(31,32)^ or clinical assessments^(33)^. VFSS monitors the entire swallowing process through sequential frames; thus, changes in any phase of swallowing can be identified objectively^(44)^. The ASHA-NOMS scale assesses the appropriate dietary program for dysphagic patients; it determines the best consistency based on clinical assessment^(45)^. The GUSS verifies the degree of dysphagia by testing with three different consistencies and indicates procedures for the appropriate consistencies for the patient's diet^(46)^.

None of the three studies addressed a specific TGS protocol, highlighting the gap in research directed at this procedure for adults with ischemic stroke. This gap highlights the need for studies with this population since this therapy is widely used in speech-language-hearing therapy. It is important to emphasize the importance of evidence-based techniques, associating the best possible evidence with clinical practice to define the best approach for the patient^(47,48)^.

On the other hand, all included articles described the TTS protocol, each according to its particularity, intervention protocol detailing the materials used, volume concentration, structures to which stimulation was applied, frequency of repetitions, and duration of the intervention. Protocol development is essential for the control of the results. Filho^(49)^ points out that the study design, research question, inclusion and exclusion criteria, sample size, and other parameters are crucial for the proper functioning of scientific research. A well-defined protocol is closely related to improved perceptions of the possibility of bias or confounding variables and the consequent elaboration of strategies to overcome them. It also helps establish accurate analyses of the results^(49)^.

Although not all articles fully described the therapeutic protocol, those that described them presented some characteristics uniformly, such as the frequency of applications per series^(31,32)^, the utensils used^(31-33)^, and the need for ice to cool the material used in the application^(31,32)^. However, other data were not as standardized, such as the cooling temperature and the time interval between removing the metal from the cooling and its application to the mucosa.

Furthermore, two articles lacked information about possible adverse effects, which may influence the assessment of risks and benefits related to the intervention and subsequent clinical decision-making, as well as the identification of safety factors for future research.

Therefore, it is essential to reflect on the application of TTS and TGS in clinical practice for adults with ischemic stroke and OD, considering the little evidence found in the literature with solid characteristics for the effective analysis of the results. Matos et al.^(49)^ conducted a review of the interventions used in the rehabilitation of dysphagia in the population with stroke and, although several procedures were analyzed, they did not include any article that addressed the techniques studied.

The need for greater specificity in the population, detailed descriptions, and complete presentation of sample data is particularly relevant, especially to obtain essential information for direct action in speech-language-hearing therapy by performing TTS and TGS with patients with ischemic stroke.

This review has some limitations. Few studies met the research eligibility criteria, and many were excluded because they restricted the generalization of the results to a broader population and not only to ischemic stroke. The limitations also include the lack of information detailing the procedures to perform robust data analysis, as well as more complete information about the population, such as the location of the lesion, time of diagnosis, NIHSS data, and other relevant characteristics about the patient’s clinical condition.

Thus, this scoping review suggests further studies with representative samples of the population with ischemic stroke and OD, with more detailed TTS/TGS therapeutic protocols, describing the training and experience of the technique applicator, more robust statistical analyses, with bias control, and analyzing the effectiveness in swallowing functioning (reintroduction of the oral route).

## CONCLUSION

The studies mapped the effect of TTS based on different methodological designs and specific measures, such as swallowing time and duration. The presentation of data on the application of the therapeutic strategy was standardized, but not all used the same pattern. No article evaluated TGS either alone or in combination with another technique.
