# Supplementary material for: Tactile, thermal and gustatory stimulation therapy in the treatment of post-stroke oropharyngeal dysphagia: a scoping review
Source: Codas. 2025 Jan 20;37(1):e20230319. doi: 10.1590/2317-1782/e20230319en (PMC11758856; doi:10.1590/2317-1782/e20230319en)
Supplement: Translation

# Translation: Terapia de estimulação tátil, térmica e gustativa no tratamento da disfagia orofaríngea pós AVCi: uma revisão de escopo

**DOI:** 10.1590/2317-1782/e20230319pt

**Authors:** Julia Matos, Rodrigo Alves de Andrade, Diego Fernando Dornelles Bilheri, Maysa Andrade Santos, Diane de Lima Oliveira, Leandro de Araújo Pernambuco, Ana Maria Furkim

**Affiliations:** 1 Programa de Graduação em Fonoaudiologia, Universidade Federal de Santa Catarina – UFSC - Florianópolis (SC), Brasil.; 2 Departamento de Fonoaudiologia, Universidade Federal de Santa Catarina – UFSC - Florianópolis (SC), Brasil.; 3 Programa de Pós-graduação em Fonoaudiologia, Universidade Federal de Santa Catarina – UFSC - Florianópolis (SC), Brasil.; 4 Departamento de Fonoaudiologia, Universidade Federal da Paraíba – UFPB - João Pessoa (PB), Brasil.

**Keywords:** Transtornos da Deglutição, Acidente Vascular Cerebral, Reabilitação do Acidente Vascular Cerebral, Sabor, Temperatura, Terapia

## Abstract

**Objetivo:**

Mapear na literatura os efeitos da estimulação tátil, térmica e/ou gustativa na disfagia orofaríngea (DO) pós AVCi.

**Método:**

Esta revisão de escopo foi conduzida segundo recomendações do PRISMA-SCR e do Joanna Briggs Institute (JBI). Registrada na Open Science Framework e desenvolvida sem restrições de idioma ou período de publicação. Foram utilizadas diferentes bases de dados e literatura cinzenta para seleção dos artigos, e os mnemônicos PCC construíram a pergunta e critérios de elegibilidade da pesquisa, assim, foram incluídos estudos clínicos envolvendo adultos (maiores de 18 anos) diagnosticados com DO pós AVCi, que receberam estimulação tátil-térmica (ETT) e/ou térmica-gustativa (ETG) e/ou estimulação tátil-térmica-gustativa para tratamento da DO e tiveram seu efeito medido a partir de exames, escalas ou avaliação clínica. A revisão foi conduzida de forma cega e independente por dois pesquisadores, e um terceiro foi consultado em casos de divergências.

**Resultados:**

Três estudos foram incluídos. Nenhum deles abordou um protocolo individual de ETG, e todos apresentaram protocolo de ETT. Os objetivos e os testes de avaliação de cada estudo foram distintos, apresentando diversas perspectivas acerca da ETT na DO; houve uniformidade na apresentação dos protocolos aplicados, frequência e materiais utilizados para a terapia.

**Conclusão:**

Os estudos mapearam o efeito da ETT a partir de diferentes desenhos metodológicos e medidas específicas; não foram encontrados artigos que avaliassem ETG isolada ou associada à outra técnica.

## INTRODUÇÃO

O Acidente Vascular Cerebral (AVC) pode ser descrito como uma interrupção ou redução brusca do fluxo sanguíneo nas vias circulatórias cerebrais, comprometendo o suprimento de oxigênio e nutrientes das células, causada por uma ruptura ou obstrução da via sanguínea^(1)^. Alguns fatores estão associados ao seu acometimento, dentre eles, a hipertensão, diabetes, colesterol LDL e triglicerídeos elevados, poluição, alto índice de gordura corporal, etilismo, baixa frequência de atividade física, tabagismo e uso de outras drogas^(2)^.

Um dos sintomas atrelado às complicações provenientes do AVC está a disfagia, caracterizada pela desorganização dos eventos sequências que envolvem o transporte de alimentos, líquidos e fluidos, da boca ao estômago, podendo, se não tratada, impactar na qualidade de vida do paciente^(3)^. A disfagia pode levar à desidratação, desnutrição, perda de peso, pneumonias aspirativas, qualidade de vida prejudicada, e à morte^(4)^. Pacientes disfágicos possuem cerca de 32% mais chances de serem alocados em setores hospitalares de cuidados mais complexos, bem como 1,7 vezes mais chance de morte em ambiente hospitalar, comparados a casos que não cursam com disfagia^(5)^.

Diante da complexidade relacionada aos protocolos de reabilitação em disfagia, Terré^(6)^ apresenta que diferentes estratégias, como manobras posturais, adaptações de dieta, estimulação tátil-térmica (ETT) e gustativa (ETG), neuroestimulação, entre outras, podem ser utilizadas considerando a necessidade do paciente, alinhando aos ganhos proporcionados pela estratégia selecionada.

Dentre as estratégias terapêuticas, a ETT e a ETG visam aumentar a sensibilidade da cavidade oral e, consequentemente, desencadear um disparo de deglutição mais rápido^(7)^. A ETT é tradicionalmente aplicada por meio de toques frios com espelho laríngeo, espátula ou espátula envolta em gaze na região do terço inferior do arco palatoglosso^(8,9)^.

A estimulação sensorial também se relaciona ao acréscimo na ativação cortical da deglutição, uma vez que melhores respostas na fase oral e faríngea a partir da ETT podem se relacionar à reorganização cortical^(10)^.

Segundo Pelletier e Lawless^(11)^, a ingestão de alimentos de sabor cítrico tem mostrado aumento substancial no número de deglutições secas espontâneas. Quando se analisam os estímulos térmico e gustativo, outros estudos apontam uma associação dos estímulos com a redução do tempo de trânsito faríngeo^(12,13)^. Estudos^(14,15)^ descrevem mudanças na deglutição após ETT em pacientes disfágicos pós AVC. Em pacientes não disfágicos, alguns estudos^(16,17)^ não observam mudanças funcionais, significativas, para a deglutição, após a estimulação tátil-térmica.

Diante do exposto, as estimulações tátil-térmica e gustativa, se mostram como uma das estratégias possíveis para reabilitação da população disfágica pós AVC, no entanto, os diferentes desenhos metodológicos, métodos de avaliação e desfechos observados dificultam a comparação e análise da efetividade da proposta terapêutica. Assim, este estudo propõe realizar uma revisão de escopo para mapear os estudos que avaliam o efeito da ETT e ETG em pacientes disfágicos pós AVCi, haja vista se tratar de um desenho de estudo que busca explorar os principais achados do tema em questão, averiguar a dimensão, o alcance e a natureza dos estudos, condensando os dados e possibilitando a partir de seus achados, identificar lacunas que direcionam perguntas a serem respondidas por revisões sistemáticas e auxiliar no desenvolvimento de uma clínica mais assertiva em seu processo de reabilitação.

A pergunta norteadora foi baseada nos elementos mnemônicos P (População), C (Conceito) e C (Contexto), sugeridos pelo guia da JBI para revisões de escopo. Logo, a População foi composta por adultos (maiores de 18 anos) com diagnóstico de disfagia orofaríngea pós Acidente Vascular Cerebral Isquêmico (AVCi); o Conceito englobou o tratamento da disfagia orofaríngea com estimulação tátil, térmica e/ou gustativa, associada ou não a outra estratégia de reabilitação e o Contexto está relacionado à reabilitação em unidade hospitalar ou ambulatorial e centro de reabilitação. Sendo assim, esta revisão de escopo pretende responder a seguinte pergunta de pesquisa: Como a literatura científica descreve os efeitos da estimulação tátil e/ou térmica e/ou gustativa em pacientes disfágicos pós AVCi atendidos em centros de reabilitação, unidades hospitalares ou ambulatoriais?

## MÉTODO

### Protocolo e registro

O protocolo desta revisão de escopo foi registrado na plataforma *Open Science Framework* sob o registro DOI 10.17605/OSF.IO/QNDKC.

### Guia de redação

Esta revisão de escopo seguiu as recomendações do *Preferred Reporting Items for Systematic reviews and Meta-Analyses extension for Scoping Reviews* (PRISMA-ScR) *Checklist* ^(18)^, assim como do *JBI Manual for Evidence Synthesis* ^(19)^.

Para a elaboração desta revisão de escopo, foram realizadas definições conceituais a priori:

*Acidente Vascular Cerebral Isquêmico:* Uma alteração na circulação sanguínea a partir de um fenômeno obstrutivo, comprometendo a passagem do sangue através das vias circulatórias cerebrais^(1)^;
*Deglutição:* A deglutição é o processo fisiológico ordenado de transferência de saliva ou conteúdo alimentar da boca ao estômago^(20)^;
*Disfagia:* uma dificuldade patológica para deglutir^(21)^;
*Reabilitação da deglutição:* alcance da estabilidade nutricional, a fim de evitar a aspiração laringotraqueal e consequências advindas desta condição^(22)^;
*Efeito*: será definido como a composição entre eficiência^(23)^, eficácia^(24)^ e segurança^(25)^;
*Estimulação Tátil Térmica (ETT):* Técnica terapêutica envolvendo a utilização do toque através de um espelho laríngeo, e da temperatura, especialmente a fria, para estimular a sensibilidade oral^(26)^.
  *Estimulação Térmica Gustativa (ETG):* Técnica terapêutica que utiliza os estímulos de temperatura e sabor com o intuito de diminuir o tempo de trânsito faríngeo^(27)^.

### Critérios de inclusão

Os critérios de inclusão para esta revisão são estudos que abordem a realização de tratamento com estimulação tátil, térmica e/ou gustativa, associada ou não a outras estratégias de reabilitação, em pacientes adultos (idade superior a 18 anos) diagnosticados com disfagia orofaríngea decorrente de AVCi e que realizaram avaliação clínica e/ou instrumental da deglutição antes e após tratamento. Foram selecionados estudos de diferentes desenhos, sem restrição de idioma e tempo de publicação.

### Critérios de exclusão

Foram excluídos artigos que não descreveram os critérios de monitoramento dos resultados pré e pós terapia para mensuração dos resultados. Estudos que analisaram a efetividade da ETTG sem separar os tipos de AVCs, sem resumo ou não encontrados para leitura completa após solicitação para os autores, revisões de literatura, publicações duplicadas, cartas, livros, conferências, resumos de conferências, artigos de opinião, guias, artigos técnicos, cartas editoriais e literatura não revisada por pares também foram excluídos.

### Fontes de informação

No dia 13 de setembro de 2023, foi realizada a busca nas bases de dados eletrônicas da saúde (Cinahl, Cochrane, Embase, Lilacs, PEDro, PUBMED/MEDLINE, PsycINFO, Scielo, Scopus e Web of Science) e na literatura cinzenta (Google Acadêmico). Em 15 de abril de 2024 foi realizada uma nova busca com finalidade de mapear possíveis estudos para a inclusão.

### Estratégia de busca

A estratégia de busca foi elaborada a partir de combinações apropriadas das palavras-chave selecionadas em uma consulta aos Descritores em Ciências da saúde (DeCS)^(28)^ e ao *Medical Subject Headings* - (MeSH)^(29)^. Termos livres foram utilizados caso fossem considerados apropriados. A estratégia de busca é apresentada no Quadro 1 de forma individualizada por base de dados.

**Quadro 1:** Descrição das estratégias de busca e o quantitativo dos estudos identificados durante a fase de seleção

**Bases de Dados**	**Estratégia de Busca**	**Estudos Identificados**
PubMed/MEDLINE	((“Stroke”[Mesh] OR “Stroke” OR “Apoplexy” OR “Brain Vascular Accident” OR “Brain Vascular Accidents” OR “CVA” OR “CVAs” OR “Cerebrovascular Accident” OR “Cerebrovascular Accidents” OR “Strokes” OR “Cerebral ictus”) AND (“Deglutition Disorders”[Mesh] OR “Deglutition Disorders” OR “Dysphagia” OR “Swallowing Disorder” OR “Swallowing Disorders” OR “Deglutition”[Mesh] OR “Deglutition”) AND (“Touch”[Mesh] OR “Touch” OR Tacti* OR “Thermosensing”[Mesh] OR “Thermosensing” OR “Thermosensings” OR “Thermic Treatment” OR “Cold” OR “Temperature” OR Thermal* OR Thermic* OR “Taste Perception”[Mesh] OR “Taste Perception” OR Gustator* OR “Taste”) AND (“Therapeutics”[Mesh] OR “Therapeutics” OR Therap* OR Treatment* OR “Rehabilitation”[Mesh] OR “Rehabilitation” OR Rehabilita*))	55
Embase	((“Stroke” OR “Apoplexy” OR “Brain Vascular Accident” OR “Brain Vascular Accidents” OR “CVA” OR “CVAs” OR “Cerebrovascular Accident” OR “Cerebrovascular Accidents” OR “Strokes” OR “Cerebral ictus”) AND (“Deglutition Disorders” OR “Dysphagia” OR “Swallowing Disorder” OR “Swallowing Disorders” OR “Deglutition”) AND (“Touch” OR Tacti* OR “Thermosensing” OR “Thermosensings” OR “Thermic Treatment” OR “Temperature” OR Thermal* OR Thermic* OR “Taste Perception” OR “Cold” OR Gustator* OR “Taste”) AND (“Therapeutics” OR Therap* OR Treatment* OR “Rehabilitation” OR Rehabilita*))	304
CINAHL	((“Stroke” OR “Apoplexy” OR “Brain Vascular Accident” OR “Brain Vascular Accidents” OR “CVA” OR “CVAs” OR “Cerebrovascular Accident” OR “Cerebrovascular Accidents” OR “Strokes” OR “Cerebral ictus”) AND (“Deglutition Disorders” OR “Dysphagia” OR “Swallowing Disorder” OR “Swallowing Disorders” OR “Deglutition”) AND (“Touch” OR Tacti* OR “Thermosensing” OR “Thermosensings” OR “Cold” OR “Temperature” OR “Thermic Treatment” OR Thermal* OR Thermic* OR “Taste Perception” OR Gustator* OR “Taste”) AND (“Therapeutics” OR Therap* OR Treatment* OR “Rehabilitation” OR Rehabilita*))	29
Cochrane Library	((“Stroke” OR “Apoplexy” OR “Brain Vascular Accident” OR “Brain Vascular Accidents” OR “CVA” OR “CVAs” OR “Cerebrovascular Accident” OR “Cerebrovascular Accidents” OR “Strokes” OR “Cerebral ictus”) AND (“Deglutition Disorders” OR “Dysphagia” OR “Swallowing Disorder” OR “Swallowing Disorders” OR “Deglutition”) AND (“Touch” OR Tacti* OR “Thermosensing” OR “Thermosensings” OR “Cold” OR “Temperature” OR “Thermic Treatment” OR Thermal* OR Thermic* OR “Taste Perception” OR Gustator* OR “Taste”) AND (“Therapeutics” OR Therap* OR Treatment* OR “Rehabilitation” OR Rehabilita*))	69
Scopus	((“Stroke” OR “Apoplexy” OR “Brain Vascular Accident” OR “Brain Vascular Accidents” OR “CVA” OR “CVAs” OR “Cerebrovascular Accident” OR “Cerebrovascular Accidents” OR “Strokes” OR “Cerebral ictus”) AND (“Deglutition Disorders” OR “Dysphagia” OR “Swallowing Disorder” OR “Swallowing Disorders” OR “Deglutition”) AND (“Touch” OR Tacti* OR “Thermosensing” OR “Thermosensings” OR “Cold” OR “Temperature” OR “Thermic Treatment” OR Thermal* OR Thermic* OR “Taste Perception” OR Gustator* OR “Taste”) AND (“Therapeutics” OR Therap* OR Treatment* OR “Rehabilitation” OR Rehabilita*))	145
Web of Science	((“Stroke” OR “Apoplexy” OR “Brain Vascular Accident” OR “Brain Vascular Accidents” OR “CVA” OR “CVAs” OR “Cerebrovascular Accident” OR “Cerebrovascular Accidents” OR “Strokes” OR “Cerebral ictus”) AND (“Deglutition Disorders” OR “Dysphagia” OR “Swallowing Disorder” OR “Swallowing Disorders” OR “Deglutition”) AND (“Touch” OR Tacti* OR “Thermosensing” OR “Thermosensings” OR “Cold” OR “Temperature” OR “Thermic Treatment” OR Thermal* OR Thermic* OR “Taste Perception” OR Gustator* OR “Taste”) AND (“Therapeutics” OR Therap* OR Treatment* OR “Rehabilitation” OR Rehabilita*))	82
Lilacs	((“Acidente Vascular Cerebral” OR “AVC” OR “AVE” OR “Acidente Cerebral Vascular” OR “Acidente Cerebrovascular” OR “Acidente Vascular Encefálico” OR “Acidente Vascular do Cérebro” OR “Acidentes Cerebrais Vasculares” OR “Acidentes Cerebrovasculares” OR “Acidentes Vasculares Cerebrais” OR “Apoplexia” OR “Derrame Cerebral” OR “Icto Cerebral” OR “Ictus Cerebral” OR “Accidente Cerebrovascular” OR “ACV” OR “Accidente Cerebral Vascular” OR “Accidente Cerebrovascular” OR “Accidente Vascular Cerebral” OR “Accidente Vascular Encefálico” OR “Accidente Vascular del Cerebro” OR “Accidentes Cerebrovasculares” OR “Apoplejía” OR “Ataque Cerebral” OR “Ataque Cerebrovascular” OR “Stroke” OR “Apoplexy” OR “Brain Vascular Accident” OR “Brain Vascular Accidents” OR “CVA” OR “CVAs” OR “Cerebrovascular Accident” OR “Cerebrovascular Accidents” OR “Strokes” OR “Cerebral ictus”) AND (“Transtornos de Deglutição” OR Disfagia* OR “Deglutição” OR “Trastornos de Deglución” OR “Deglución” OR “Deglutition Disorders” OR “Dysphagia” OR “Swallowing Disorder” OR “Swallowing Disorders” OR “Deglutition”) AND (“Tato” OR “Tátil” OR “Táteis” OR “Sensação Térmica” OR “Tratamento Térmico” OR “Estimulação gustativa” OR “Estimulação tátil-térmica” OR Térmic* OR “Percepção Gustatória” OR Gustativ* OR “Tacto” OR “Sensación Térmica” OR “Tratamiento Térmico” OR “Percepción del Gusto” OR “Gusto” OR “Temperatura” OR “Touch” OR Tacti* OR “Thermosensing” OR “Thermosensings” OR “Cold” OR “Temperature” OR “Thermic Treatment” OR Thermal* OR Thermic* OR “Taste Perception” OR Gustator* OR “Taste”) AND (“Terapêutica” OR Terapêutica* OR Terapia* OR Tratamento* OR “Reabilitação” OR Reabilita* OR Tratamiento* OR “Rehabilitación” OR “Therapeutics” OR Therap* OR Treatment* OR “Rehabilitation” OR Rehabilita*))	6
PEDro	Stroke* Dysphag*	128
Scielo	((“Acidente Vascular Cerebral” OR “AVC” OR “AVE” OR “Acidente Cerebral Vascular” OR “Acidente Cerebrovascular” OR “Acidente Vascular Encefálico” OR “Acidente Vascular do Cérebro” OR “Acidentes Cerebrais Vasculares” OR “Acidentes Cerebrovasculares” OR “Acidentes Vasculares Cerebrais” OR “Apoplexia” OR “Derrame Cerebral” OR “Icto Cerebral” OR “Ictus Cerebral” OR “Accidente Cerebrovascular” OR “ACV” OR “Accidente Cerebral Vascular” OR “Accidente Cerebrovascular” OR “Accidente Vascular Cerebral” OR “Accidente Vascular Encefálico” OR “Accidente Vascular del Cerebro” OR “Accidentes Cerebrovasculares” OR “Apoplejía” OR “Ataque Cerebral” OR “Ataque Cerebrovascular” OR “Stroke” OR “Apoplexy” OR “Brain Vascular Accident” OR “Brain Vascular Accidents” OR “CVA” OR “CVAs” OR “Cerebrovascular Accident” OR “Cerebrovascular Accidents” OR “Strokes” OR “Cerebral ictus”) AND (“Transtornos de Deglutição” OR Disfagia* OR “Deglutição” OR “Trastornos de Deglución” OR “Deglución” OR “Deglutition Disorders” OR “Dysphagia” OR “Swallowing Disorder” OR “Swallowing Disorders” OR “Deglutition”) AND (“Tato” OR “Tátil” OR “Táteis” OR “Sensação Térmica” OR “Tratamento Térmico” OR “Estimulação gustativa” OR “Estimulação tátil-térmica” OR Térmic* OR “Percepção Gustatória” OR Gustativ* OR “Tacto” OR “Sensación Térmica” OR “Tratamiento Térmico” OR “Percepción del Gusto” OR “Gusto” OR “Temperatura” OR “Touch” OR Tacti* OR “Thermosensing” OR “Thermosensings” OR “Cold” OR “Temperature” OR “Thermic Treatment” OR Thermal* OR Thermic* OR “Taste Perception” OR Gustator* OR “Taste”) AND (“Terapêutica” OR Terapêutica* OR Terapia* OR Tratamento* OR “Reabilitação” OR Reabilita* OR Tratamiento* OR “Rehabilitación” OR “Therapeutics” OR Therap* OR Treatment* OR “Rehabilitation” OR Rehabilita*))	2
PsycINFO	((“Stroke” OR “Apoplexy” OR “Brain Vascular Accident” OR “Brain Vascular Accidents” OR “CVA” OR “CVAs” OR “Cerebrovascular Accident” OR “Cerebrovascular Accidents” OR “Strokes” OR “Cerebral ictus”) AND (“Deglutition Disorders” OR “Dysphagia” OR “Swallowing Disorder” OR “Swallowing Disorders” OR “Deglutition”) AND (“Touch” OR Tacti* OR “Thermosensing” OR “Thermosensings” OR “Cold” OR “Temperature” OR “Thermic Treatment” OR Thermal* OR Thermic* OR “Taste Perception” OR Gustator* OR “Taste”) AND (“Therapeutics” OR Therap* OR Treatment* OR “Rehabilitation” OR Rehabilita*))	5
Google Acadêmico	Português:	300
	(“Acidente Vascular Cerebral” OR “AVC” OR “AVE” OR “Acidente Vascular Encefálico”) AND (Disfagia* OR “Deglutição”) AND (“Tato” OR “Tátil” OR Táteis OR Térmic* OR “Gustatória” OR Gustativ*) AND (Terapêutica* OR Terapia* OR Tratamento* OR Reabilita*)	
	Espanhol:	
	(“Accidente Cerebrovascular” OR “ACV” OR “Accidente Vascular Encefálico”) AND (Disfagia* OR “Deglución”) AND (“Tacto” OR Térmic* OR “Gusto” OR “Temperatura”) AND (Terapia* OR Tratamiento* OR “Rehabilitación”)	
	Inglês:	
	(“Stroke” OR “Strokes” OR “CVA” OR “CVAs” OR “Cerebrovascular Accident”) AND (“Dysphagia” OR “Swallowing Disorder” OR “Deglutition”) AND (“Touch” OR Tacti* OR “Thermosensing” OR Thermal* OR Thermic* OR Gustator* OR “Taste”) AND (Therap* OR Treatment* OR Rehabilita*)	

### Processo de seleção de evidências

Para manter a avaliação dos textos seguindo os critérios pré-estabelecidos e um padrão de concordância entre as análises, a calibração foi inter revisores. Para isso, foi aplicado o Coeficiente de Concordância de Kappa como medida de qualidade de ajuste com o intuito de viabilizar dados de maior qualidade que possibilitam melhor avaliação das coincidências e divergências obtidas durante a calibração. Ao fim da calibração, alcançou-se um nível quase perfeito de coeficiente de concordância entre os revisores (Kappa > 90).

Para realizar a calibração, um examinador padrão (M.A.S), que participou da orientação acerca da pesquisa e uso da plataforma de gestão *Rayyan Sistematic* ^(30)^, conduziu todo o processo de treinamento dos revisores independentes, ambos fonoaudiólogos (D.F.D.B/J.M). O treinamento foi realizado com quarenta e dois artigos e, após, os revisores independentes (D.F.D.B/J.M) realizaram as etapas às cegas. As decisões sobre eventuais discordâncias foram resolvidas por consenso a fim de garantir a qualidade dos processos, e com a participação do terceiro revisor (A.M.F) quando necessário.

Todos os artigos identificados foram incluídos no *Rayyan Systematic* e, após a calibração, foram analisados. A seleção dos artigos foi realizada em duas etapas por dois revisores (D.F.D.B/J.M), de forma independente e às cegas: Na primeira etapa, foram analisados os títulos e resumos das citações coletadas nos bancos de dados, e selecionados os estudos potencialmente relevantes. Na segunda etapa, os revisores realizaram a leitura integral dos artigos seguindo os critérios de elegibilidade e excluindo os estudos que não atendiam aos critérios.

As decisões sobre eventuais conflitos foram resolvidas entre os revisores por consenso para garantir a qualidade dos processos, e com a participação do terceiro revisor (A.M.F) quando necessário.

### Extração de dados

Para aumentar a confiabilidade dos dados extraídos sobre ETT e ETG, foram incluídos estudos que realizaram avaliação clínica e/ou instrumental da deglutição antes e após tratamento. Além disso, para comparar os resultados pré e pós tratamento, foram coletados os dados de média, desvio padrão e p valor. Toda a extração foi realizada por dois avaliadores, de forma independente e às cegas, e um terceiro avaliador foi consultado quando necessário.

Foram extraídos os dados relacionados a autoria, ano, país, desenho de estudo, objetivo do estudo, dados de diagnóstico (dados do NIHSS, tempo de Ictus e local da avaliação), perfil da amostra (quantidade de participantes, idade, sexo e localização/lado do AVCi), informações dos profissionais responsáveis pela terapia, testes clínicos ou instrumentais para avaliação pré e pós, os protocolos de ETT, ETG ou do tratamento combinado, os resultados e, por fim, os dados de possíveis eventos adversos.

#### Síntese dos resultados

Os resultados da busca nas bases de pesquisa foram organizados em um diagrama de fluxo; e os dados extraídos, em uma tabela. Os dados foram organizados de acordo com classificações estabelecidas pelos juízes e, quando não disponíveis de forma quantitativa nos estudos, sintetizados a partir da descrição oferecida no corpo do texto dos artigos.

## RESULTADOS

O diagrama de fluxo da seleção de estudos está apresentado na Figura 1. Foram identificados 820 estudos nas bases de dados e, após a remoção das duplicatas, foram lidos título e resumo dos 542 estudos restantes. 26 estudos foram elegíveis para leitura integral durante a fase dois, e três destes foram incluídos nesta revisão de escopo^(31-33)^.

**Figura 1:** Fluxograma da seleção de estudos

Os estudos incluídos foram publicados nos anos de 1991^(31)^, 1996^(32)^ e 2021^(33)^, sendo o mais recente na Coreia do Sul^(33)^ e os dois restantes nos Estados Unidos^(31,32)^. Quanto aos desenhos de estudo, o primeiro é um desenho experimental de sujeito único *(ABAB design)* ^(31)^ replicado com sete outros sujeitos, o segundo é um estudo cruzado *(crossover)* ^(32)^, e o terceiro é um estudo experimental randomizado controlado^(33)^.

Os estudos se propuseram a verificar a influência térmica na deglutição, se a influência se prolongaria e se testes base poderiam prever a resposta do sujeito à estimulação térmica^(31)^; apresentar dados da variabilidade na duração da deglutição e os efeitos de curto prazo da aplicação térmica^(32)^; verificar, a partir da estimulação com capsaicina em baixa temperatura, o efeito na disfagia, no nível alimentar, no risco de aspiração ou penetração e no estado nutricional^(33)^.

Acerca dos dados clínicos apresentados pelos estudos, dois deles incluíram pacientes com histórico de múltiplos AVCis^(31,32)^; dois apresentaram os dados da ferramenta *National Institutes of Health Stroke Scale (NIHSS)* ^(31,33)^; um artigo não apresentou o tempo de ictus^(33)^, um apresentou em dias^(31)^ e outro em semanas^(32)^; e apenas dois artigos informaram o local onde as avaliações ocorreram^(32,33)^.

Os estudos analisaram amostras predominantemente masculinas, com n entre 7 (sete) e 43 participantes, sendo dois estudos com análise intragrupo^(31,32)^ e um intergrupo^(33)^. Um artigo classificou o AVC a partir do lado acometido^(33)^ (com maior porcentagem do lado direito no grupo intervenção e no esquerdo para o grupo controle), enquanto os demais descreveram a partir do local de acometimento^(31,32)^.

Quanto a avaliação da deglutição, dois artigos realizaram avaliação instrumental com videofluoroscopia da deglutição (VFD)^(31,32)^ e um artigo utilizou o protocolo *Gugging Swallowing Screen* (GUSS) para avaliação clínica da deglutição e o *Measurement System Swallowing Scale* (ASHA-NOMS) para avaliar o nível dietético^(33)^.

Nenhum dos estudos apresentou informações específicas sobre a formação e experiência dos profissionais responsáveis pela terapia^(31-33)^.

Todos os três artigos descreveram os materiais utilizados e a frequência de aplicação. Os três artigos também apresentaram o protocolo do procedimento: dois artigos realizaram a aplicação de um espelho laríngeo resfriado de tamanho 00, nos pilares anteriores das fauces, três vezes de cada lado, e, após, solicitaram a deglutição de um volume^(31,32)^; o terceiro artigo realizou a aplicação em mucosa orofaríngea de uma solução de capsaicina, água e espessante, utilizando um abaixador de língua envolto em gaze, com posterior aplicação de solução de capsaicina em cavidade oral^(33)^. Todos abordaram a ETT^(31-33)^ e nenhum apresentou ETG no estudo. Um artigo relatou a ausência de efeitos adversos a partir da intervenção^(33)^ e dois artigos não relataram possíveis eventos^(31,32)^.

A Tabela 1 apresenta os dados das amostras, protocolo, dados de avaliação e resultados encontrados nos estudos selecionados, todos referindo-se à terapia de ETT.

**Tabela 1:** Dados extraídos a partir dos estudos selecionados

Autor, ano (País)	Desenho do Estudo	Objetivo do estudo	Diagnóstico (Dados NIHSS, Tempo de Ictus, Local da avaliação)	Perfil da Amostra		Testes Utilizados para Avaliação da Deglutição e Durante o Tratamento	Profissional responsável pela Terapia	Protocolo ETT ou ETG	Tratamento Combinado	Resultados	Eventos Adversos
				Grupo Intervenção	Grupo Controle						
Rosenbek et al.^(31)^ 1991 (EUA)	Desenho experimental de sujeito único (*ABAB design*) com replicação em outros 7 sujeitos	1: A estimulação térmica diária influencia a deglutição de líquidos?; 2: As influências se mantêm após um mês do fim do tratamento?; 3: Testes de base podem prever a resposta da estimulação térmica?	AVCi múltiplos; NIHSS 9 a 23; Tempo de ictus: mais recente 19 dias, mais antigo relatado 5110 dias; Local: NI	**7 homens; Idade** 65 a 77 anos; **Local do AVC** 2 em ponte, 1 em cerebelo, 2 na artéria cerebral média esquerda, 2 na artéria cerebral média direita		**VFD**: uma vez pré terapia, quatro vezes durante (1x/semana), e uma vez um mês pós terapia	Clínico	Aplicação de um espelho laríngeo resfriado, de tamanho 00, nos pilares anteriores das fauces, três vezes de cada lado. Após, o participante deglutiu 3cc de água em colher ou pipeta ou recebeu um pequeno volume de pedaços de gelo.	NI	**Medidas de duração: IMPB** (s) (média-DP): 0.15/0.43 - 0.27/0.73 IMPB (s) (média-DP) **CBABPRM** (s) (média-DP): 0.03/0.08 - 0.06/0,10; **IEMH** (s) (média-DP): 0.03/0.08 - 0.06/0.06; **PEMH** (s) (média-DP): 0.02/0.11 - 0.02/0.12; **UMMEH** (s) (média-DP): 0.03/0.22 - 0.05/0.19; **PMAH** (s) (média-DP): 0.02/0.03 - 0.02/0.04; **UMPMAH** (s) (média-DP): 0.02/0.08 - 0.02/0.05; **HRR** (s) (média-DP): 0.10/0.16 - 0.019/0.20; **EESA** (s) (média-DP): 0.01/0.03 - 0.02/0.02; **EESF** (s) (média-DP): 0.03/0.06 - 0.03/0.06; **SBEES** (s) (média-DP): 0.03/0.05 - 0.03/0.04. **DST 1ª/2ª/3ª deglutição (s): Sujeito 1:** 0.72/0.61/1.80; **Sujeito 2:** 0.53/1.37/0.25; **Sujeito 3:** 0.51/-0.17/9.64; **Sujeito 4:** 0.33/0.22/0.56; **Sujeito 5:** 2.28/0.65/0.20; **Sujeito 6:** -0.38/0.29/0.41; **Sujeito 7:** 0.18/-0.13/- ; **DST por grupo, (s): 63-67 anos: Média (DP):** -0.07 (0.27); **70-76 anos: Média (DP):** 0.11 (0.45).	NI
Rosenbek et al.^(32)^ 1996 (EUA)	Estudo cruzado	Fornecer dados de variabilidade nos parâmetros duracionais da deglutição medidos objetivamente, realizados por pacientes disfágicos secundários a AVC, e examinar os efeitos de curto prazo da aplicação térmica nessas mesmas medidas duracionais.	AVCi múltiplos NIHSS NI Tempo ictus: <1 a 172 semanas Local: Hospital	**22 participantes;** Idade 55 a 81 anos (média 67.3). **Local do AVC** 2 em cerebelo, 5 em membro posterior da cápsula interna, 3 em ponte, 3 na junção ponto-mesencefálica esquerda, 5 em gânglios da base, 4 em lobo parietal esquerdo.		**VFD:** realizada durante as dez deglutições solicitadas	Clínico experiente	Aplicação de um espelho laríngeo resfriado, de tamanho 00, nos pilares anteriores das fauces, três vezes de cada lado. A estimulação em cada lado foi realizada em menos de seis segundos após a retirada do espelho do gelo. Após a estimulação, os pacientes realizaram a deglutição de um bolo.	NI	**DST: Não tratados: Média (DP):** 1.25 (0.47) **Mediana:** 0.42 **Tratados: Média (DP):** 0.89 (0.38) **Mediana:** 0.38 **Diferença: Média (DP):** -0.35 (0.19) **Mediana:** -0.15 ***p*** 0.046; **TSD: Não tratados: Média (DP):** 2.96 (.50) **Mediana:** 2.22 **Tratados: Média (DP):** 2.44 (.40) **Mediana:** 2.00 **Diferença: Média (DP):** -0.52 (.20) **Mediana:** -0.22 ***p*** 0.005	NI
Bae e Lee^(33)^ 2021 (Korea)	Estudo experimental randomizado controlado antes e depois	Determinar o efeito da estimulação sensorial orofaríngea com capsaicina de baixa temperatura no distúrbio de deglutição, nível alimentar, pneumonia aspirativa e estado nutricional em pacientes internados na unidade de terapia intensiva de AVC com disfagia.	NIHSS média (DP) - 9.65 (6.52); Tempo de Ictus - NI; 40 avaliações em Hospital, 3 avaliações em Ambulatório	**21 participantes; Idade** 70.76 (9.19); **Homens** 11 (52.4%), **Mulheres** 10 (47.6%); **Lado do AVCi: Esquerdo** 8 (38.1); **Direito** 12 (57.1); **Bilateral** 0; **Múltiplo** 1 (4.8)	**22 participantes; Idade** 72.86 (11.83); **Homens** 13 (59.1%), **Mulheres** 9 (40.9%); **Lado do AVCi: Esquerdo** 12 (54.5), **Direito** 6 (27.3), **Bilateral** 4 (18.2), **Múltiplo** 0	**GUSS:** 1 vez antes da intervenção e no 3º e 7º dias de intervenção. Avaliado o nível de disfagia; **ASHA-NOMS:** utilizado para avaliar o nível dietético pré terapia e nos dias 3 e 7 de intervenção.	NI	Foram diluídos 2g de capsaicina em pó a 100mL de água engarrafada - solução com concentração de 150uM/L. Foi incluído um espessante *(Viscoup)*, um pacote por 100mL. Um abaixador de língua envolvido com gaze foi embebido na solução e o pesquisador esfregou suave e rapidamente a mucosa orofaríngea, dando leves tapinhas na região. Após, 1mL de solução de capsaicina foi pedido em uma seringa de 2cc, a agulha foi removida e o conteúdo foi aplicado na cavidade oral do paciente. A intervenção foi aplicada duas vezes ao dia antes das refeições, durante sete dias. O grupo controle recebeu apenas água, sem adição de capsaicina, ao mesmo tempo, método e número de vezes.	NI	**Disfagia: Grupo experimental: Início:** 11,76±4,84; **3º dia:** 14,14±5,23; **7º dia:** 16,38±4,84 (χ^2^ =34,74, p < 0,001)**; Grupo controle: Início:** 10,95±7,20**; 3º dia:** 11,68±6,85; **7º dia:** 12,59±7,08 (χ^2^ = 5,41, p = 0,067); **Nível dietético: Grupo experimental: Início:** 3,43 ± 1,47; **3º dia:** 4,19±1,81; **7º dia:** 4,81±1,81 (χ^2^ =25,29, p < 0,001), **Grupo controle: Início:** 3,36±2,15; **3º dia:** 3,45±1,81; **7º dia:** 3,77±2,22 (χ^2^=4,51, p = .105); **Pneumonia aspirativa: Dia 3:** sem casos relatados; **Dia 7: Grupo controle:** 6 (27,3%); **Grupo experimental:** 2 (9,5%); Taxa de incidência sem diferença clara entre grupos (x^2^=2,23, p = 0,240).	Sem eventos adversos

Legenda: *NIHSS* = Escala do AVC; *ETT* = Estimulação tátil-térmica; *ETG* = Estimulação térmica-gustativa; *AVCi* = Acidente vascular cerebral isquêmico; *NI* = não informado; *VFD* = Videofluoroscopia da deglutição; *GUSS* = Gugging Swallowing Screen; *ASHA-NOMS* = Measurement System Swallowing Scale; *DST* = Duração do estágio transitório; *TSD* = duração total da deglutição; *IMPB* = Início do movimento posterior do bolo; *CBABPRM* = A cabeça do bolo atinge a borda posterior do ramo mandibular; *IEMH* = Início da elevação máxima do hióide; *PEMH* = Primeira elevação máxima do hióide; *UMMEH* = Último momento da máxima elevação do hióide; *PMAH* = Posição mais anterior do hióide; *UMPMAH* = Último momento do posicionamento mais anterior do hióide; *HRR* = Hióide retorna ao repouso; *EESA* = Esfíncter esofágico superior se abre; *EESF* = Esfíncter esofágico superior se fecha; *SBEES* = Saída do bolo do esfíncter esofágico superior; *DP* = desvio padrão; *x^2^* = teste qui-quadrado; *p* = p-valor; *cc* = centímetro cúbico; *g* = gramas; *s* = segundos; *mL* = mililitros; *µM/L* = micrômetro/litro

## DISCUSSÃO

As abordagens em ETT e ETG são propostas frequentemente utilizadas na terapia de pacientes com disfagia orofaríngea pós AVCi. Compreender os efeitos de tais técnicas é imprescindível para garantir intervenções que de fato manifestem melhora no quadro do paciente. Assim, essa revisão de escopo, com o intuito de verificar os efeitos das terapias de ETT e ETG, buscou apresentar um panorama dos estudos que abordam estas técnicas na população adulta com AVCi e disfagia atendidos em centros de reabilitação, hospitais e ambulatórios.

Dentre os estudos selecionados, nenhum dos artigos abordou a terapia de ETG isolada ou associada à ETT. Todos os três estudos incluídos abordaram exclusivamente a ETT^(31-33)^.

Apesar de diferentes estudos abordarem a ETT e ETG entre as possibilidades terapêuticas para a DO, foi predominante, ao longo da seleção de artigos, a presença de estudos que analisaram amostras heterogêneas, pertencendo ao mesmo grupo de análise, participantes com AVC isquêmico e hemorrágico (AVCh)^(34-38)^, sendo esta característica encontrada dentro dos grupos controle e intervenção^(34,36)^.

Ao analisar a eficiência e eficácia de diversos procedimentos terapêuticos, Silva^(39)^ faz uma crítica à pouca especificidade nas amostras relacionadas às disfagias neurogênicas, visto que há distinções entre o comprometimento de cada doença e, sem uma amostra homogênea, há pouca robustez na coleta de evidência, e tal crítica vai ao encontro dos dados obtidos nesta revisão, uma vez que estudos foram excluídos por avaliarem o efeito da ETT/ETG considerando AVCi e AVCh em uma mesma amostra comparativa. Portanto, considerando a variação de comprometimento do AVCi e do AVCh relacionados à disfagia e a frequência destes^(40,41)^, ambas as populações devem ser abordadas independentemente.

Os objetivos de cada estudo incluído foram distintos entre si, e, nesse sentido, diversos fatores relacionados à metodologia foram influenciados, podendo-se destacar a variação de dias de intervenção e o plano terapêutico elaborado por cada artigo: um dos estudos teve como objetivo analisar a influência da ETT diária na deglutição de líquidos e verificar o padrão da deglutição a longo prazo (um mês)^(31)^; outro artigo buscou analisar os efeitos da ETT a curto prazo^(32)^; e um terceiro artigo buscou determinar o efeito da aplicação de capsaicina em baixa temperatura em quatro aspectos - disfagia, nível dietético, pneumonia aspirativa e estado nutricional^(33)^. É possível verificar diversas perspectivas acerca da aplicação da ETT e, apesar da pouca quantidade de estudos incluídos, há como obter informações e identificar lacunas para maiores investigações em pesquisas futuras.

O tamanho da amostra nos estudos incluídos, que variou entre sete a 43 participantes, evidencia a necessidade de estudos com populações numericamente mais representativas para se atingir resultados com maior solidez, considerando que AVCi afeta cerca de 7.6 milhões de pessoas por ano globalmente^(42)^, e tem como uma das principais sequelas a disfagia orofaríngea, que pode afetar cerca de 50% dos indivíduos^(43)^.

Além disso, outros dados importantes acerca da amostra, como o tempo de ictus, porcentagem de pessoas do sexo masculino e feminino nos grupos, região afetada pelo AVCi, valores de *NIHSS* e local das avaliações não apresentaram padronização em sua apresentação, não foram incluídos em sua totalidade ou não foram informados. Os estudos incluídos, de modo geral, também não descreveram informações referentes à formação e à experiência prática dos profissionais que realizaram as avaliações e a terapia. Tais dados podem comprometer a comparabilidade entre estudos, a análise das informações e a interpretação destas por outros pesquisadores, além da validade dos resultados.

Outro aspecto relevante é a escolha de diferentes testes para avaliação e análise dos efeitos na disfagia orofaríngea. As distintas formas de classificação se basearam em avaliações de imagem^(31,32)^ ou avaliações clínicas^(33)^. A videofluoroscopia da deglutição (VFD) permite acompanhar todo o processo da deglutição através de quadros sequenciais e, assim, identificar alterações em quaisquer fases da deglutição de forma objetiva^(44)^. A ASHA-NOMS é uma escala utilizada para avaliar o programa dietético adequado para o paciente disfágico; tem relação à determinação da melhor consistência a partir da avaliação clínica^(45)^. A escala GUSS permite verificar o grau da disfagia a partir do teste com três consistências diferentes e indicar condutas relacionadas às consistências adequadas para a alimentação do paciente^(46)^.

Ademais, não houve estudos incluídos que abordassem protocolo específico de ETG, destacando a lacuna em pesquisas direcionadas a esse procedimento para a população adulta com AVCi. Esta lacuna traz a necessidade de desenvolvimento de estudos com esta população, uma vez que se trata de uma terapêutica amplamente utilizada na clínica fonoaudiológica. É importante ressaltar a importância da técnica baseada em evidência, associando a melhor evidência possível aliada à prática clínica para definir a melhor conduta para o paciente^(47,48)^.

Quanto ao protocolo de ETT, todos os artigos incluídos descreveram, cada um de acordo com sua particularidade, um protocolo de intervenção detalhando os materiais utilizados, concentração dos volumes, estruturas as quais a estimulação foi aplicada, frequência de repetições e duração da intervenção. A elaboração de protocolos são essenciais para o controle dos resultados obtidos. Filho^(49)^ aponta que o delineamento do tipo de estudo, da questão a ser pesquisada, dos critérios de inclusão e exclusão, tamanho da amostra, entre outros parâmetros, são cruciais para o funcionamento adequado de uma pesquisa científica; um protocolo bem definido possui estrita relação com percepções aprimoradas de possibilidade de viés ou de variáveis confundidoras e consequente elaboração de estratégias para superá-las, e, ainda, auxilia no estabelecimento de análises acuradas acerca dos resultados obtidos^(49)^.

Embora nem todos os artigos tenham descrito completamente o protocolo terapêutico, aqueles que o mencionaram mostraram uma uniformidade em sua apresentação em relação a algumas características, como a frequência de aplicações por série^(31,32)^, a apresentação dos utensílios utilizados^(31-33)^ e a necessidade de gelo para resfriamento do material utilizado na aplicação^(31,32)^. No entanto, outras informações não receberam essa mesma padronização, tais quais a temperatura de resfriamento e o intervalo de tempo entre a retirada do metal do resfriamento e sua aplicação na mucosa.

Ainda, considerando a ausência de informações em dois artigos acerca dos possíveis efeitos adversos, a falta destes dados pode influenciar na avaliação de riscos e benefícios relacionados à intervenção e posterior tomada de decisão clínica, assim como na identificação de fatores de segurança para pesquisas futuras.

Diante do exposto, é fundamental a reflexão sobre a aplicação da ETT e ETG na prática clínica para a população adulta com AVCi e DO, considerando a pouca evidência encontrada na literatura com características sólidas para a análise efetiva dos resultados. Matos et al.^(49)^ realizaram uma revisão das intervenções utilizadas na reabilitação em disfagia na população com AVC e, apesar de diversos procedimentos serem analisados, não houve inclusão de artigos que abordassem as técnicas estudadas.

A necessidade de maior especificidade na população, descrições detalhadas e apresentação completa dos dados da amostra, torna-se particularmente relevante, especialmente, para se obter informações essenciais à atuação direta na terapia fonoaudiológica realizando ETT e ETG com o paciente acometido por AVCi.

Esta revisão apresenta algumas limitações. A escassez de estudos que cumpriam os critérios de elegibilidade da pesquisa, neste caso muitos estudos foram excluídos por restringir a generalização dos resultados para uma população mais ampla e não apenas para AVCi. Além disso, observa-se como limitações a falta de informações detalhando os procedimentos para realizar uma análise robusta dos dados, bem como informações mais completas sobre a população, como por exemplo, local da lesão, tempo de diagnóstico, dados do *NIHSS* e outras características relevantes sobre a clínica dos indivíduos.

Assim, esta revisão de escopo sugere o desenvolvimento de estudos com amostras representativas da população com AVCi e DO, com protocolos terapêuticos de ETT/ETG mais detalhados, com descrição de treinamento e experiência do aplicador da técnica, análises estatísticas mais robustas, com controle de vieses e analisando a efetividade na funcionalidade da deglutição (reintrodução da via oral).

## CONCLUSÃO

Os estudos mapearam o efeito da ETT a partir de diferentes desenhos metodológicos e medidas específicas, como tempo e duração das deglutições. Observou-se a padronização na apresentação de dados relacionados à aplicação da estratégia terapêutica, porém nem todos apresentaram o mesmo padrão. Não foram encontrados artigos que avaliassem a ETG isolada ou associada à outra técnica.
